# Assembly of Mitochondrial Genomes Using Nanopore Long‐Read Technology in Three Sea Chubs (Teleostei: Kyphosidae)

**DOI:** 10.1111/1755-0998.14034

**Published:** 2024-10-15

**Authors:** J. Antonio Baeza, Jeremiah J. Minish, Todd P. Michael

**Affiliations:** ^1^ Department of Biological Sciences Clemson University Clemson South Carolina USA; ^2^ Smithsonian Marine Station at Fort Pierce Smithsonian Institution Fort Pierce Florida USA; ^3^ Departamento de Biología Marina, Facultad de Ciencias del Mar Universidad Católica del Norte Coquimbo Chile; ^4^ The Plant Molecular and Cellular Biology Laboratory Salk Institute for Biological Studies La Jolla California USA

**Keywords:** long‐read sequencing, nanopore, rudderfish

## Abstract

Complete mitochondrial genomes have become markers of choice to explore phylogenetic relationships at multiple taxonomic levels and they are often assembled using whole genome short‐read sequencing. Herein, using three species of sea chubs as an example, we explored the accuracy of mitochondrial chromosomes assembled using Oxford Nanopore Technology (ONT) Kit 14 R10.4.1 long reads at different sequencing depths (high, low and very low or genome skimming) by comparing them to ‘gold’ standard reference mitochondrial genomes assembled using Illumina NovaSeq short reads. In two species of sea chubs, *Girella nigricans* and *Kyphosus azureus*, ONT long‐read assembled mitochondrial genomes at high sequencing depths (> 25× whole [nuclear] genome) were identical to their respective short‐read assembled mitochondrial genomes. Not a single ‘homopolymer insertion’, ‘homopolymer deletion’, ‘simple substitution’, ‘single insertion’, ‘short insertion’, ‘single deletion’ or ‘short deletion’ were detected in the long‐read assembled mitochondrial genomes after aligning each one of them to their short‐read counterparts. In turn, in a third species, *Medialuna californiensis*, a 25× sequencing depth long‐read assembled mitochondrial genome was 14 nucleotides longer than its short‐read counterpart. The difference in total length between the latter two assemblies was due to the presence of a short motif 14 bp long that was repeated (twice) in the long read but not in the short‐read assembly. Read subsampling at a sequencing depth of 1× resulted in the assembly of partial or complete mitochondrial genomes with numerous errors, including, among others, simple indels, and indels at homopolymer regions. At 3× and 5× subsampling, genomes were identical (perfect) or almost identical (quasiperfect, 99.5% over 16,500 bp) to their respective Illumina assemblies. The newly assembled mitochondrial genomes exhibit identical gene composition and organisation compared with cofamilial species and a phylomitogenomic analysis based on translated protein‐coding genes suggested that the family Kyphosidae is not monophyletic. The same analysis detected possible cases of misidentification of mitochondrial genomes deposited in GenBank. This study demonstrates that perfect (complete and fully accurate) or quasiperfect (complete but with a single or a very few errors) mitochondrial genomes can be assembled at high (> 25×) and low (3–5×) but not very low (1×, genome skimming) sequencing depths using ONT long reads and the latest ONT chemistries (Kit 14 and R10.4.1 flowcells with SUP basecalling). The newly assembled and annotated mitochondrial genomes can be used as a reference in environmental DNA studies focusing on bioprospecting and biomonitoring of these and other coastal species experiencing environmental insult. Given the small size of the sequencing device and low cost, we argue that ONT technology has the potential to improve access to high‐throughput sequencing technologies in low‐ and moderate‐income countries.

## 1 Introduction

Complete mitochondrial genomes have become markers of choice for examining phylogenetic relationships at multiple taxonomic levels both in vertebrate and invertebrate taxa (Veldsman et al. 2020; Chak, Barden, and Baeza 2020; Chak, Baeza, and Barden 2021; Ennis et al. 2021; Gutiérrez et al. 2023, among many others). The use of entire or mostly complete mitochondrial genomes for phylogenetic inference is appealing in large part because of their high mutation rate in comparison with most nuclear markers and their ‘nearly‐neutral’ rate of molecular evolution (Ballard and Rand 2005; Bernt et al. 2013; but see Galtier et al. 2009 for exceptions). Moreover, given that mitochondrial inheritance is maternal‐only (clonal), the mitochondrial genome functions as a single nonrecombining locus (see Ladoukakis and Zouros 2017 and references therein for exceptions). Lastly, multiple copies of a mitochondrial genome exist within each metazoan cell. Therefore, the extraction and sequencing of mitochondrial DNA is straightforward relative to nuclear DNA.

During the last decade, next‐generation sequencing (NGS) technologies (i.e., BGI and Illumina short reads) have been routinely used for the assembly of entire mitochondrial genomes using either high‐ and low‐coverage whole genome sequencing (WGS) or genome skimming (very low‐coverage [= or < 1×] WGS) that can be enriched or not for mitochondrial markers (Veldsman et al. 2020; Chak, Baeza, and Barden 2021; Ennis et al. 2021; Gutiérrez et al. 2023). Although with enough resources (personnel, funding, NGS platform availability), short‐read assembled mitochondrial genomes can be obtained in hours or days, most often a project to assemble mitochondrial genomes is time consuming, taking from weeks to years. In fact, it could be argued that BGI and/or Illumina short‐read platforms are not the ideal solution for studies that require the speedy recovery of molecular markers (including mtDNA) such as real‐time genomic disease surveillance (e.g., Faria et al. 2018) or in situ detection of mislabelling and/or misidentification of legal or illegal biological commodities (e.g., shark fins, rhinoceros horns, ivory, ornamental plants and animals) in the supply chain (Baeza and Behringer 2017).

Third generation sequencing technology (i.e., long reads from Pacific Biosciences [PacBio] and Oxford Nanopore Technologies [ONT] platforms) capable of sequencing DNA fragments regularly longer than animal mitochondrial genomes (i.e., ~10–20 kbp and up to 1–2 Mbp—Jain et al. 2018) can be considered an alternative to short‐read NGS platforms for assembling entire mitochondrial genomes in relatively short times. Importantly, however, the first platforms developed for sequencing long DNA fragments exhibited relatively high initial sequence error rates (ONT = 5%–15%; PacBio = 11%–15%) that were much higher than those achieved by contemporary short‐read sequencing platforms (< 0.3%) (Jünemann et al. 2013). Still, remarkable progress has been achieved during the last few years with new long‐read platform models and chemistries that, ONT and PacBio claim (see www.nanoporetech.com and www.pacb.com, respectively), have resulted in substantial decreases in the initial sequencing error rate. Independent benchmarking of such improved platforms and chemistries (as claimed by companies developing long‐read sequencing platforms) is rare but of utmost importance as benchmarking information can assist with the optimisation of benchwork and bioinformatics workflows for the de novo assembly of relatively short (i.e., mitochondrial) or long (nuclear) genomes using long‐read sequencing exclusively.

Only a few but an increasing number of studies have relied on ONT or PacBio long reads exclusively for the assembly—de novo—of entire mitochondrial genomes (Johri et al. 2019, 2020; Franco‐Sierra and Díaz‐Nieto 2020; Malukiewicz et al. 2021; Baeza 2020; Baeza and García‐De León 2022, among a few others). Perhaps more importantly, only a limited number of the few studies that have used ONT and PacBio long‐read technology exclusively to assemble mitochondrial genomes have reported how accurate they are and benchmarked the long‐read assembled mitochondrial genomes with short‐read assembled mitochondrial genomes, ideally generated from the same individual (Baeza 2020; Baeza and García‐De León 2022; Uliano‐Silva et al. 2023). Among them, Baeza (2020) and Baeza and García‐De León (2022) reported the assembly of complete but not perfect mitochondrial genomes using ONT long reads exclusively. A comparison of short‐read and ONT long‐read assembled mitochondrial genomes indicated that discordance between the assemblies was mostly due to indels of variable lengths at the flanks of homopolymer regions (Baeza 2020; Baeza and García‐De León 2022).

In this study, we are interested in revisiting the accuracy problem of ONT long‐read for the assembly of mitochondrial genomes. Specifically, we formally tested if it is possible to assemble ‘perfect’ (complete and fully accurate) mitochondrial genomes using the latest nanopore long‐read sequencing technology at high (> 25×), low (3–5×) and very low (1×, genome skimming) sequencing depths. To achieve our aim, following Baeza (2020) and Baeza and García‐De León (2022), we benchmarked the quality (i.e., accuracy) of the long‐read assembled genomes using the latest ONT technology by comparing them to ‘gold’ standard mitochondrial genomes assembled using Illumina short reads that were retrieved from the same individuals. As a model system, we used three shallow water marine fishes from the Southern California Bight located within the northeast Pacific basin. The three species were the Opaleye *Girella nigricans*, Zebra‐perch *Kyphosus azureus* and Halfmoon *Medialuna californiensis* sea chubs. All three species belong to the family Kyphosidae, are often targeted by recreational fishers in the west coast of the USA, are exposed to increased environmental local and global challenges (Shanks et al. 2020), and only a few genomic resources exist for them (Minich et al. 2023).

**FIGURE 1:**
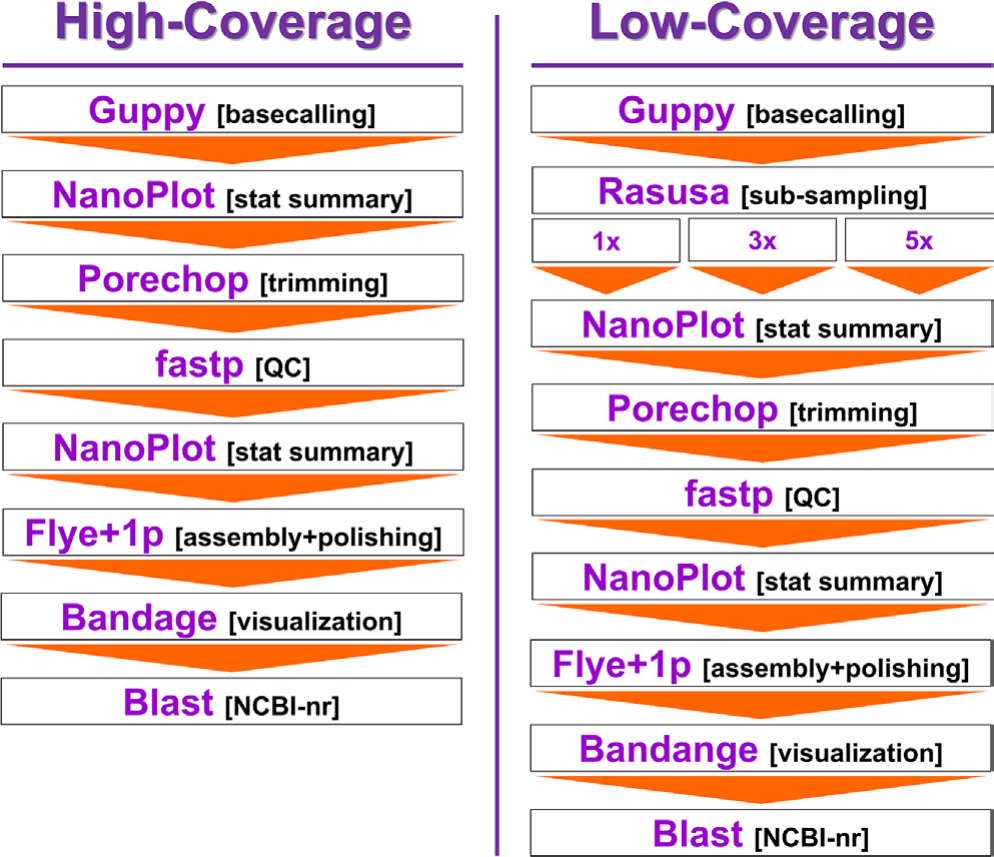
Bioinformatics pipeline to assemble the mitochondrial chromosome of sea chubs using nanopore long reads at high (> 25×), low (3–5×) and very low (1×, genome skimming) sequencing depth.

To assemble the ONT long‐read mitochondrial genomes, we employed a modified bioinformatics pipeline specifically developed for the de novo assembly of mitochondrial genomes using long reads exclusively (Figure 1) (Baeza 2020; Baeza and García‐De León 2022). Also following Baeza (2020) and Baeza and García‐De León (2022), the sequence accuracy of the long‐read assemblies was explored with multiple metrics including completeness, identity and coverage plus a detailed quantitative analysis of error type in long‐read assemblies. We subsampled the original ONT data sets (> 25×) to explore the efficiency of the used bioinformatic pipelines to assemble mitochondrial genomes at low (3–5×) and very low (1×, genome skimming) coverage sequencing depths. Lastly, we described the mitochondrial genomes in detail following recommendations in Baeza (2020) and we explored with the newly assembled mitochondrial genomes the phylogenetic position of the three studied species using a mitophylogenomics strategy.

## 2 Methods

### 2.1 DNA Genomic Data Sets

We utilised the data set recently generated by Minich et al. (2023) to assemble the mitochondrial genome of the different fish species using ONT long reads exclusively and explore their accuracy. Details on collection of the different fish specimens, genomic DNA (gDNA) extraction, library preparation, sequencing and raw signal (FAST5 files) basecalling are provided in the aforementioned study. We note that Minich et al. (2023) used the ligation kit V14 chemistry for library preparation and sequenced on the Oxford Nanopore Technologies Promethion24 using the R10.4.1 flow cells. Basecalling was done using the on‐instrument software MinKnow version 22.1 (Oxford Nanopore Technologies). These were the most advanced hardware and chemistry developed by ONT at the time.

In parallel to the long‐read Nanopore sequencing data, short‐read Illumina data were generated from the same DNA samples for the three species of fish. Using approximately 20 ul of whole blood from each fish sample, gDNA was extracted using the NEB Monarch HMW DNA extraction kit ‘for cells and blood’ (Cat# T3050L, New England Biolabs). gDNA was quantified using the Qubit fluorometer Broad Range dsDNA kit (Cat#Q32850, Thermo fisher).

At least 100 ng of DNA isolated from whole blood of fish was used as input material to the PCR‐free, ligation‐based, Illumina library preparation using the Collibri ES kit (Thermo Fisher, Cat# A38605024). This kit utilises an enzymatic shearing rather than mechanical so not up front shearing is performed on the DNA. The DNA libraries were then submitted to the Salk Institute NGS Core and were sequenced on a NovaSeq 6000. The Salk Institute NGS Core is supported by funding from NIH‐NCI CCSG: P30 CA01495, NIH‐NIA San Diego Nathan Shock Center P30 AG068635, the Chapman Foundation, and the Helmsley Charitable Trust.

### 2.2 Illumina Reads Mitochondrial Genomes Assembly and Annotation

A total of 153,741,118, 159,084,722 and 159,862,515 pair‐end (PE) reads belonging to *G*. *nigricans*, *K*. *azureus* and *M*. *californica* were generated by the sequencing facility (SRA Accession number SRR28380136 [*G*. *nigricans*], SRR28380135 [*K*. *azureus*] and SRR28380134 [*M*. *californiensis*]), respectively, and were used to assemble the mitochondrial genome of the three studied species. Mitochondrial genomes of each of the three studied fish species were ‘de novo’ assembled using the program GetOrganelle v1.6.4 (Jin et al. 2020). The mitochondrial genome of the cofamilial *Kyphosus cinerascens* available in NCBI's GeneBank (AP011061) was used as a ‘seed’ for all assemblies that were run with k‐mer sizes of 21, 55, 85 and 115. Next, the web servers MITOS2 (Donath et al. 2019), as implemented in the Galaxy web platform (The Galaxy Community 2022), and MitoFish v3.63 (http://mitofish.aori.u‐tokyo.ac.jp/—Zhu et al. 2023) were used to annotate each newly assembled mitochondrial genome. In line with Baeza and García‐De León (2022), these short‐read assemblies represent the ‘gold standard’ for each of the studied species and were used as references for benchmarking the accuracy of the de novo assembled genomes using nanopore long reads exclusively at different sequencing depth.

### 2.3 ONT Long Reads Mitochondrial Genomes Assembly at High Sequencing Depth

Mitochondrial genomes for the same three specimens were ‘de novo’ assembled using high‐coverage (> 25×) ONT long reads exclusively using a strategy modified from Baeza (2020) and Baeza and García‐De León (2022) (Figure 1). First, a total of 4,936,492 (28.4 Gbp), 2,261,350 (25.5 Gbp) and 9,089,031 (43.5 Gbp) ONT reads (in FASTQ format) belonging to *G*. *nigricans*, *K*. *azureus* and *M*. *californica*, were retrieved from NCBI's GenBank (SRA Accession number SRR24529091, SRR24529093 and SRR24529092, respectively). Next, we used the program Porechop (https://github.com/rrwick/Porechop) to trim adapters from the ends of each read and to split sequences with internal adapters into two. Then, we used the software fastp (Chen et al. 2018) to quality‐filter the reads and retain only those sequences with Q‐score ≥ 6. Next, we filtered out reads shorter and greater than 5,000 and 20,000, respectively. Lastly, we assembled de novo the mitochondrial genomes of each of the studied species using the program Flye 2.8‐0 (Kolmogorov et al. 2019). We did not attempt to assembly mitochondrial genomes with the programs Canu (Koren et al. 2017) and Unicycler (Wick et al. 2017) considering that previous studies have demonstrated more accurate mitochondrial genomes assemblies with Flye compared to Unicycler and Canu (see Baeza 2020; Baeza and García‐De León 2022). In the pipeline Flye, we polished the assembled contigs with the program Flye polisher using a single polishing iteration taking into account that previous studies indicated that a single iteration results in more accurate mitochondrial genome assemblies compared to a larger number of iterations (i.e., 5 and 10 iterations, see Baeza 2020, Baeza and García‐De León 2022). Lastly, we blasted (BLAST+ 2.5.0) all assembled contigs to the mitochondrion nucleotide nonredundant database in NCBI's GenBank and calculated the statistical significance of the matches.

Based on the mitochondrial genome length of previously published species available in GenBank, we predicted that a circular sequence ~16.5–16.6 kpb long would be observed among the contigs and annotated as fish mitochondrial sequences if our pipeline was successful to assemble a circularised (complete) mitochondrial chromosome of the three studied species.

We note that we did not employ the software Medaka (https://github. com/nanoporetech/medaka) as a final ‘extra polishing’ step in our pipeline. The program Medaka utilises neural networks to a pileup of individual nanopore reads against a draft assembly to create a new final consensus sequence (Baeza 2020; Baeza and García‐De León 2022) and it is recommended as a final step when assembling genomes with ‘noisy’ ONT reads. We did not apply this extra step considering the high quality of the assembled mitochondrial genomes using the pipeline Flye, exclusively (see Section 3).

### 2.4 ONT Long Reads Mitochondrial Genomes Assembly at Low Sequencing Depths

ONT sequencing coverage depth (coverage per bp) to assemble the mitochondrial genomes of *G*. *nigricans*, *K*. *azureus* and *M*. *californica*, were 28.38× (28.4 Gbp), 25.46× (25.5 Gbp) and 43.52× (43.5 Gbp), respectively. To explore the accuracy of ONT long‐read mitochondrial genomes at low and very low‐coverage sequencing depth, we randomly subsampled each data set to 1× (very low, genome skimming), 3× (low) and 5× (low) using the program Rasusa (Hall 2022). For subsampling, we assumed a fish nuclear genome size of 1 Gb in all three studied species (see https://www.genomesize.com/). Subsampled reads were subjected to the same assembly pipeline above excluding the step in which reads shorter and longer than 5,000 and 20,000 bp were filtered out. Any partial or complete mitochondrial genome assembled using the low‐coverage data sets was also annotated using the workflow described above.

### 2.5 Accuracy of Long‐Read Assembled Mitochondrial Genomes

We compared the short‐read to all the long‐read assembled mitochondrial genomes to evaluate the accuracy of the long‐read assembled mitochondrial genomes employing four different metrics: (i) number of assembled contigs identified as of mitochondrial origin, (ii) total assembly length (all contigs), (iii) coverage and (iv) identity. The latter proxy for accuracy was measured as patristic distance (p‐distance) after aligning the long‐read to the short‐read assembled reference genome using the program Muscle (Edgar 2004) as implemented in the program MEGA X (Kumar et al. 2018). A p‐distance value equal to zero denotes identical long‐read and short‐read reference mitochondrial genomes with increasing values of p‐distance indicating lower long‐read assembled mitochondrial genome accuracy. Furthermore, as in Baeza (2020) and Baeza and García‐De León (2022), we quantified long‐read assembly error after alignment with the short‐read assembly. We classified errors as single, double, triple, quadruple, quintuple, sextuple or septuple ‘homopolymer insertions’ or ‘homopolymer deletions’ if the error added or removed, respectively, a single, two, three, four, five, six or seven bases from a homopolymer (i.e., multiple consecutive appearances of the same nucleotide) regions two or more bases in length (Baeza 2020; Baeza and García‐De León 2022). Errors observed in the long‐read assembly that did not fit with the aforementioned categories were classified as ‘simple substitution’, ‘single insertion’, ‘short insertion’, ‘single deletion’ and ‘short deletion’ (Baeza 2020; Baeza and García‐De León 2022).

### 2.6 Annotation of Mitochondrial Genome Assemblies

Each high‐coverage long‐read assembled mitochondrial genome was also annotated with the pipelines MITOS2 and MitoFish and described in detail following recommendations in Baeza (2020). In short, the nucleotide usage of each mitochondrial genome was estimated using the software MEGA X. The overall codon usage and relative synonymous codon usage of the different mitochondrial protein‐coding genes (PCGs) were computed, respectively, using the web server Sequence Manipulation Suite (https://www.bioinformatics.org/sms2/codon_usage.html—Stothard 2000) and the EZcodon tool in the web server EZmito (http://ezmito.unisi.it/ezcodon—Cucini et al. 2021). The secondary structures of tRNA genes predicted using the program MITFI (Jühling et al. 2012), as implemented in MITOS2 (Bernt, Donath, et al. 2013), in the studied mitochondrial genomes were visualised using the online tool Forna (http://rna.tbi.univie.ac.at/forna/; Kerpedjiev, Hammer, and Hofacker 2015). Lastly, microsatellites and short tandem repeats present in the mitochondrial CRs were detected using the programs Microsatellite Repeats Finder (http://insilico.ehu.es/mini_tools/microsatellites/—Bikandi et al. 2004) and Tandem Repeats Finder (https://tandem.bu.edu/trf/trf.html—Benson 1999), respectively while the secondary structure of the same regions was predicted using the web server RNAfold (http://rna.tbi.univie.ac.at/cgi‐bin/RNAWebSuite/RNAfold.cgi—Gruber et al. 2008) with attention to the presence of ‘stem‐and‐loop’ in this region.

### 2.7 Phylomitogenomics of the Family Kyphosidae

To determine the phylogenetic position of the studied species within the family Kyphosidae, the three high‐coverage long‐read assembled mitochondrial genomes together with other nine annotated mitochondrial genomes available in NCBI's GenBank belonging to the family Kyphosidae were used for maximum likelihood (ML) phylogenetic inference. Additional mitochondrial genomes belonging to the closely related families Kuhliidae (*n* = 3 species), Oplegnathidae (*n* = 4 mitochondrial genomes belonging to 2 species) and Terapontidae (*n* = 10 mitochondrial genomes belonging to 7 species) were used as outgroups during the analysis. For the analysis, we first extracted all 13 PCG nucleotides from the different mitochondrial genomes, translated each extracted sequence to amino acids using MEGA X, and aligned each amino acid sequences for each PCG with the program Clustal Omega (Siervers et al. 2011). Poorly aligned regions in each PCG data set (aligned amino acids) were removed with the software trimAl (Capella‐Gutiérrez, Silla‐Martínez, and Gabaldón 2009). Next, we used the program ProtTest (Darriba et al. 2011) to find the best‐fitting models of sequence evolution for each PCG. Finally, the previously concatenated and partitioned PCG amino acid data set was used to perform a ML analysis in the software IQ‐TREE version 1.6.10 (Nguyen et al. 2015). One thousand bootstrap pseudoreplications were conducted to estimate support for each node in the ML phylogenetic tree.

## 3 Results

### 3.1 Mitochondrial Genome Assembly Using Short Reads

**FIGURE 2:**
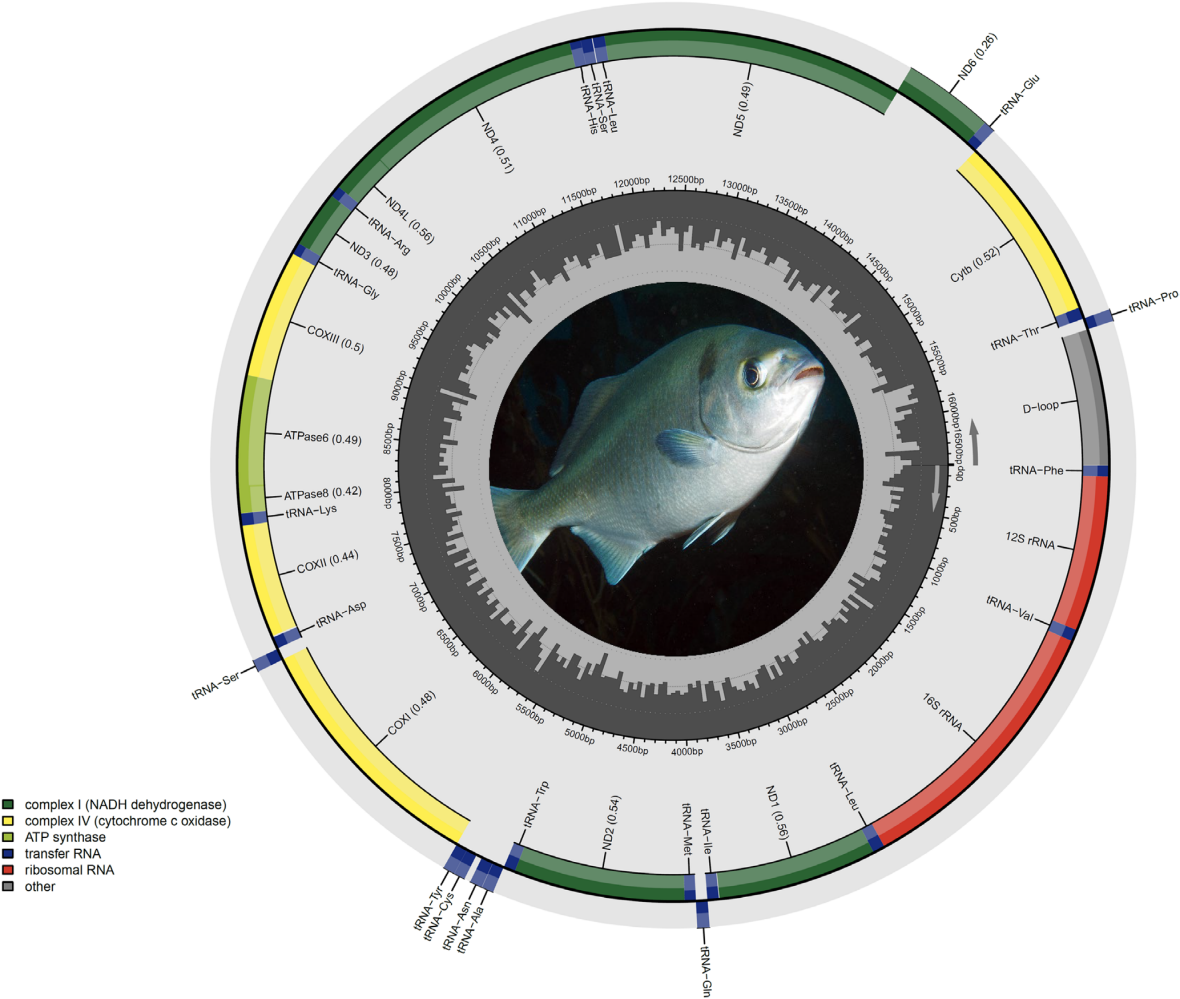
Circular map of the mitochondrial genome in *Medialuna californiensis*. The map is annotated and depicts 13 protein‐coding genes (PCGs), 22 transfer RNA (tRNA) genes, 2 ribosomal RNA genes (rrnS [12S ribosomal RNA] and rrnL [16S ribosomal RNA]) and a single putative control region. Mitochondrial gene composition and organisation was identical to that of *Girella nigricans* and *Kyphosus azureus*. Photograph of *Medialuna californiensis* by Scott Johnson (used with permission).

Using Illumina short reads, the pipeline GetOrganelle assembled complete (circularised) mitochondrial genomes for *Girella nigricans*, *Kyphosus azureus* and *Medialuna californiensis* with an average mitochondrial genome coverage of 111.7×, 176.8× and 162.9×, respectively. The complete mitochondrial genome of *G*. *nigricans*, *K*. *azureus* and *M*. *californiensis* (GeneBank accession numbers PP437477, PP437478 and PP437477, respectively) were 16,502, 16,507 and 16,498 bp in length, respectively (Figure 2).

### 3.2 Mitochondrial Genome Assembly at High Sequencing Depth Using Long Reads

Using all ONT long reads, the program Flye assembled circularised mitochondrial genomes for the three studied species with average coverages of 39× (*G*. *nigricans*), 94× (*K*. *azureus*) and 43× (*M*. *californiensis*). The long‐read assembled mitochondrial genomes of *G*. *nigricans* and *K*. *azureus* were identical (patristic distance = 0) to their respective short‐read assembled mitochondrial genomes: not a single, double, triple, quadruple, quintuple, sextuple or septuple ‘homopolymer insertion’ or ‘homopolymer deletion’, ‘simple substitution’, ‘single insertion’, ‘short insertion’, ‘single deletion’ or ‘short deletion’ were detected after aligning the two assembled mitochondrial genomes (ONT versus Illumina short read) for each of these two species (Table 1). In turn, the long‐read assembled mitochondrial genome of *M*. *californiensis* was 16,512 bp; 14 nucleotides longer than the short‐read assembled mitochondrial genome. The difference in total length between the compared assemblies was due to the presence of a short motif 14 bp long (5′—AAT GTT CTG GTG GG—3′), starting at position 1,664 in the assembled mitochondrial genomes, that was repeated twice in the long‐read assembled mitochondrial genome (Table 1).

**TABLE 1:** Accuracy metrics for different mitochondrial genome de novo assemblies using nanopore long reads exclusively in three species of sea chubs.

Sequencing depth	Contig	Length	Coverage	p‐distance	Errors
*Girella nigricans*					
1×	Partial	11,629	3×	0.002754	51
3×	Circular	16,514	5×	0.000258	11
5×	Circular	16,502	8×	0.000000	0
28.38×	Circular	16,502	111.7×	0.000000	0
*Kyphosus zinereus*					
1×	Partial	16,497	4×	0.001155	44
3×	Circular	16,508	19×	0.000000	0
5×	Circular	16,507	19×	< 0.00001	1
25.46×	Circular	16,508	176.8×	0.000000	0
*Medialuna californiensis*					
1×	Circular	16,532	3×	0.001031	31
3×	Circular	16,499	10×	< 0.00001	1
5×	Circular	16,498	12×	0.000000	0
43.52×	Circular	16,512	162.9×	< 0.00001	1

### 3.3 Mitochondrial Genome Assembly at Low and Very Low (Genome Skimming) Sequencing Depth

**FIGURE 3:**
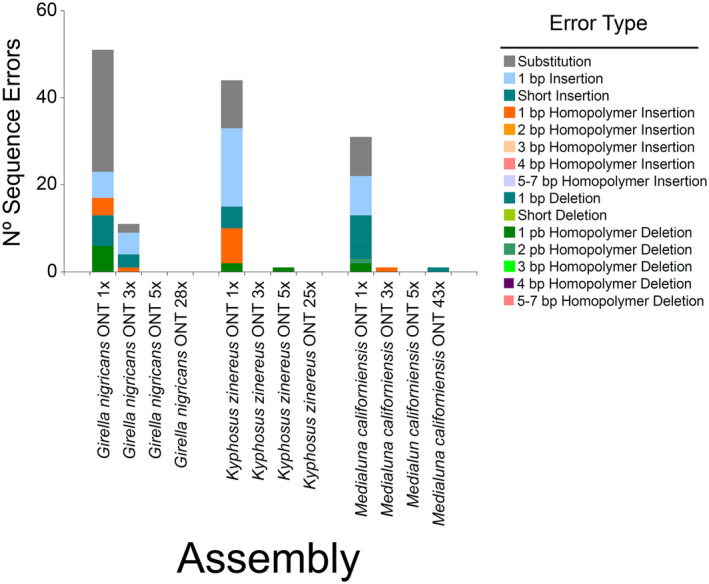
Sequence errors exhibited by mitochondrial genomes de novo assembled using ONT long reads at different sequencing coverage depths in three species of sea chubs. Benchmarking of all long‐read assemblies was performed against the Illumina short‐read assembly (‘gold’ standard).

In *G*. *nigricans*, a total of 173,612, 522,559 and 869,067 reads were subsampled by the program Rasusa to decrease sequencing coverage depth to 1×, 3× and 5×, respectively. At 1× (genome skimming, very low sequencing depth), a single partial mitochondrial genome 11,629 bp long (4,873 bp shorter than that assembled using high‐coverage ONT and Illumina sequencing) was assembled with an average coverage of 3× and exhibited a total of 51 errors, most of them single‐nucleotide substitutions followed by single insertions, single deletions, single homopolymer insertions and single homopolymer deletions, among a few others (Table 1 and Figure 3). At 3× sequencing depth, a complete (circularised by the program Flye) mitochondrial genome, 16,514 bp long (12 bp longer than that assembled at high sequencing depth), was assembled with an average coverage of 5×. The latter mitochondrial genome contained a total of 11 errors, most of them single‐nucleotide inserts and single substitutions, among a few others (Figure 3). Lastly, at 5×, the assembled mitochondrial genome was ‘perfect’; identical to those assembled at high sequencing depth ONT and Illumina short reads (Table 1).

In *K*. *azureus*, a total of 88,508, 265,849 and 445,228 reads were subsampled to decrease sequencing coverage depth to 1×, 3× and 5×, respectively. At 1× sequencing depth, a nearly complete mitochondrial genome (not circularised by the Flye assembler), 16,497 bp long (11 bp shorter than that assembled at high sequencing depth) was assembled with a coverage of 4×. A total of 44 errors, most of them simple insertions or single insertions at homopolymer regions and simple substitutions were observed in the latter mitochondrial genome (Figure 3). At 3×, the assembled mitochondrial genome (coverage = 19x) was ‘perfect’; identical to those assembled with high sequencing depth ONT and Illumina short reads. In turn, at 5×, the assembled mitochondrial genome (19×) was identical to those assembled with high sequencing depth ONT and Illumina reads with the exception of a single deletion in a long (11 bp) T‐homopolymer region (Table 1 and Figure 3).

Lastly, in *M*. *californiensis*, a total of 209,425, 627,502 and 1,044,559 reads were subsampled, respectively, to decrease sequencing coverage depth to 1×, 3× and 5×. At all sequencing depths, Flye circularised the assembled mitochondrial genomes that were 16,532 (coverage = 3×), 16,499 (10×) and 16,498 (12×) bp long, and 13 bp shorter, 20 bp longer and 14 bp longer, respectively, than the 16,512 bp long assembled using all available ONT reads. At 1×, the fully assembled mitochondrial genome exhibited a total of 44 errors, most of them simple indels and simple substitutions, among a few other errors (Figure 3). At 3×, the assembled ONT mitochondrial genome was identical to that assembled with Illumina reads with the exception of a single insertion in a long (5 bp) A‐homopolymer region. Lastly, at 5×, the assembled mitochondrial genome was identical to that assembled with Illumina short reads. All these three assembled mitochondrial genomes lacked the short motif 14 bp long that was repeated in the high‐coverage ONT long‐read assembly (Table 1 and Figure 3).

### 3.4 Annotation of Mitochondrial Genomes Assembled at High Coverage

Each of the newly assembled mitochondrial genomes contained 13 protein‐coding genes (PCGs), 2 ribosomal RNA genes (rRNAs), 22 transfer RNA genes (tRNAs) and a relatively long (approx. 850 bp) noncoding control region (CR). All PCGs except *nad6*, the two rRNA genes and 14 tRNA genes were located on one (positive, +) while the remaining 8 tRNA genes were encoded on the negative strand (Figure 2). All three newly assembled mitochondrial genomes were A + T rich and little variability in nucleotide composition was observed among them (Table 2).

**TABLE 2:** Nucleotide usage of the complete mitochondrial genome, rDNA genes, and control region (CR) assembled using ONT long reads in three species of fishes from California.

Sample	A%	T%	C%	G%	A + T%	Length
mtDNA						
*Girella nigricans*	28.92	26.47	28.38	16.23	55.39	16,502
*Kyphosus azure*	27.78	25.44	30.09	16.69	53.21	16,507
*Medialuna californiensis*	27.78	27.01	28.31	16.89	54.79	16,512
rrnS gene						
*Girella nigricans*	32.21	20.53	26.00	21.26	52.74	950
*Kyphosus azure*	30.91	20.09	26.18	21.97	51.84	951
*Medialuna californiensis*	30.35	20.44	25.92	23.29	50.79	949
rrnL gene						
*Girella nigricans*	34.31	21.06	24.78	19.86	55.37	1667
*Kyphosus azure*	31.80	21.22	25.70	21.28	53.02	1654
*Medialuna californiensis*	31.98	21.62	24.73	21.68	53.59	1670
Control Region						
*Girella nigricans*	32.49	28.88	22.50	16.13	61.37	831
*Kyphosus azure*	33.29	31.15	19.74	15.81	64.45	841
*Medialuna californiensis*	32.61	31.42	19.30	16.67	64.03	834

**FIGURE 4:**
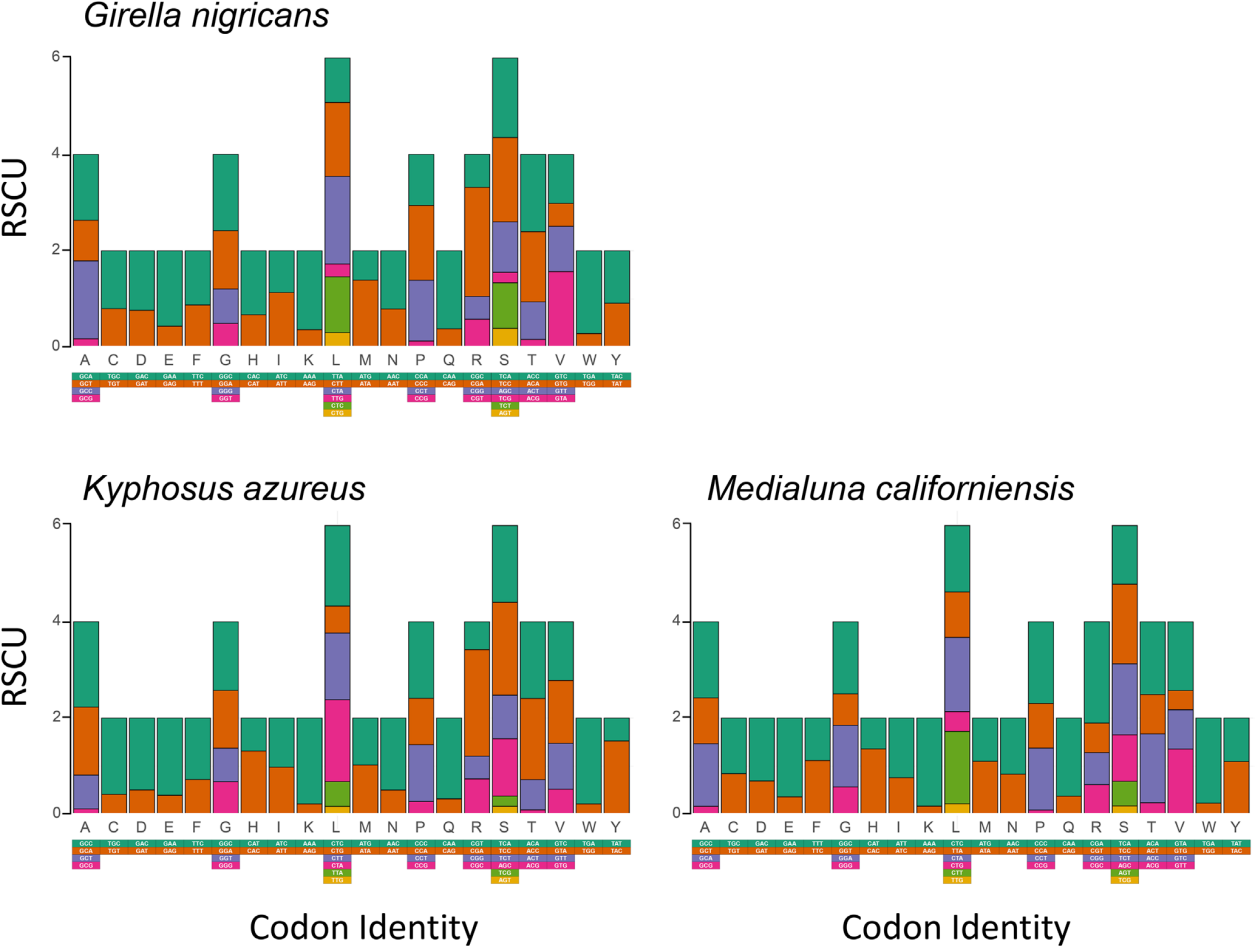
Relative synonymous codon usage in mitochondrial genomes of sea chubs.

In the PCGs of all studied mitochondrial genomes, codons were not used evenly. The most frequently used codons (in *G*. *nigricans*, *K*. *azureus* and *M*. *californiensis*) were CTA (Leu, *N* = 199, 185, and 170), CTT (Leu, *N* = 188, 151 and 166), CTC (Leu, *N* = 127, 182 and 152), ATT (Ile, *N* = 155, 126 and 164) and TTC (Phe, *N* = 131, 153 and 127). In turn, not used or least frequently used codons (other than the complete stop codons TAA and TAG) included AGG (Ser, not used), AGA (Ser, not used), CCG (Pro, *N* = 7, 15 and 5), CGG (Arg, *N* = 9, 9 and 13) and TCG (Ser, *N* = 9, 9 and 7). Similarly, synonymous codons were not used evenly in the different PCGs of the studied mitochondrial genomes, and low disparity in RSCU was observed in the PCGs of the studied mitochondrial genomes (Figure 4).

**FIGURE 5:**
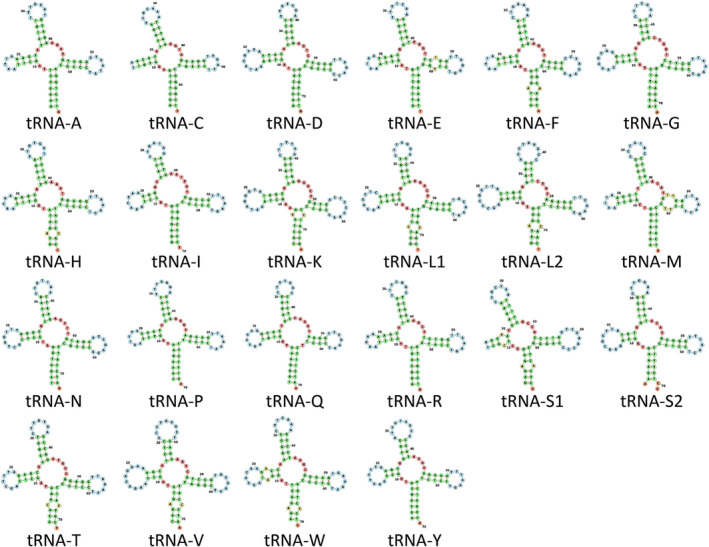
Secondary structure of tRNA genes in the species of sea chubs.

tRNA genes in the mitochondrial genomes of *G*. *nigricans*, *K*. *azureus* and *M*. *californiensis* varied between 66 (tRNA‐C) and 74 (tRNA‐K and tRNA‐L2) in length. All tRNA genes exhibited the standard ‘cloverleaf’ secondary structure as predicted by MitFI except tRNA‐Ser1 that lacked the D‐arm loop (Figure 5).

In the mitochondrial genome of *G*. *nigricans*, *K*. *azureus* and *M*. *californiensis*, the rrnS gene was 950, 951 and 949 bp in length while the rrnL gene was 1657, 1654 and 1670 bp in length, respectively. The two rDNA genes are located close to each other, between tRNA‐Phe and tRNA‐Leu and separated by tRNA‐Val. The two genes were A + T rich (Table 2). Minimal differences in nucleotide usage of the two rRNA gene was observed among the three studied species.

The CR is 831, 841 and 834 bp long in *G*. *nigricans*, *K*. *azureus* and *M*. *californiensis*, respectively. In the three species, the CR is flanked by tRNA‐Phe and tRNA‐Pro. The nucleotide usage of all CRs is A + T rich with an A + T content greater than that estimated for the entire mitochondrial genomes (Table 2). A total of 10, 10 and 8 microsatellites were found in the CR of *G*. *nigricans*, *K*. *azureus* and *M*. *californiensis*, respectively, Microsatellites invariably comprised dinucleotide motifs, most of them A + T rich, and repeated between three and five times (Tables S1–S3). The CR of *G*. *nigricans* contains 2 A + T rich tandem repeats, with consensus length 17 (5′‐ACA TAT ATG TAT TAT CA‐3′) and 20 (5′‐ATA CAT TAA TAG TAA TCA AC‐3′) repeated 5.4 and 4.7 times, respectively. The CR of *K*. *azureus* also contains 2 A + T rich tandem repeats with consensus length 17 (5′‐ATG TAA TTT AAA CAT TA‐3′) and 21 (5′‐CAT TAA TTG ATA TTC CAG GCA‐3′) repeated 4.8 and 2.7 times, respectively. Lastly, the CR of *M*. *californiensis* contains a single A + T rich tandem repeat with consensus length 23 (5′‐CAA ATT AGA GTA TAA TAA TTA AA‐3′) repeated 2.3 times. Numerous ‘hairpin’ secondary structures were present along the entire length of each CR as predicted by the web server RNAfold (Figures S1–S3).

### 3.5 Phylomitogenomics of the Family Kyphosidae

**FIGURE 6:**
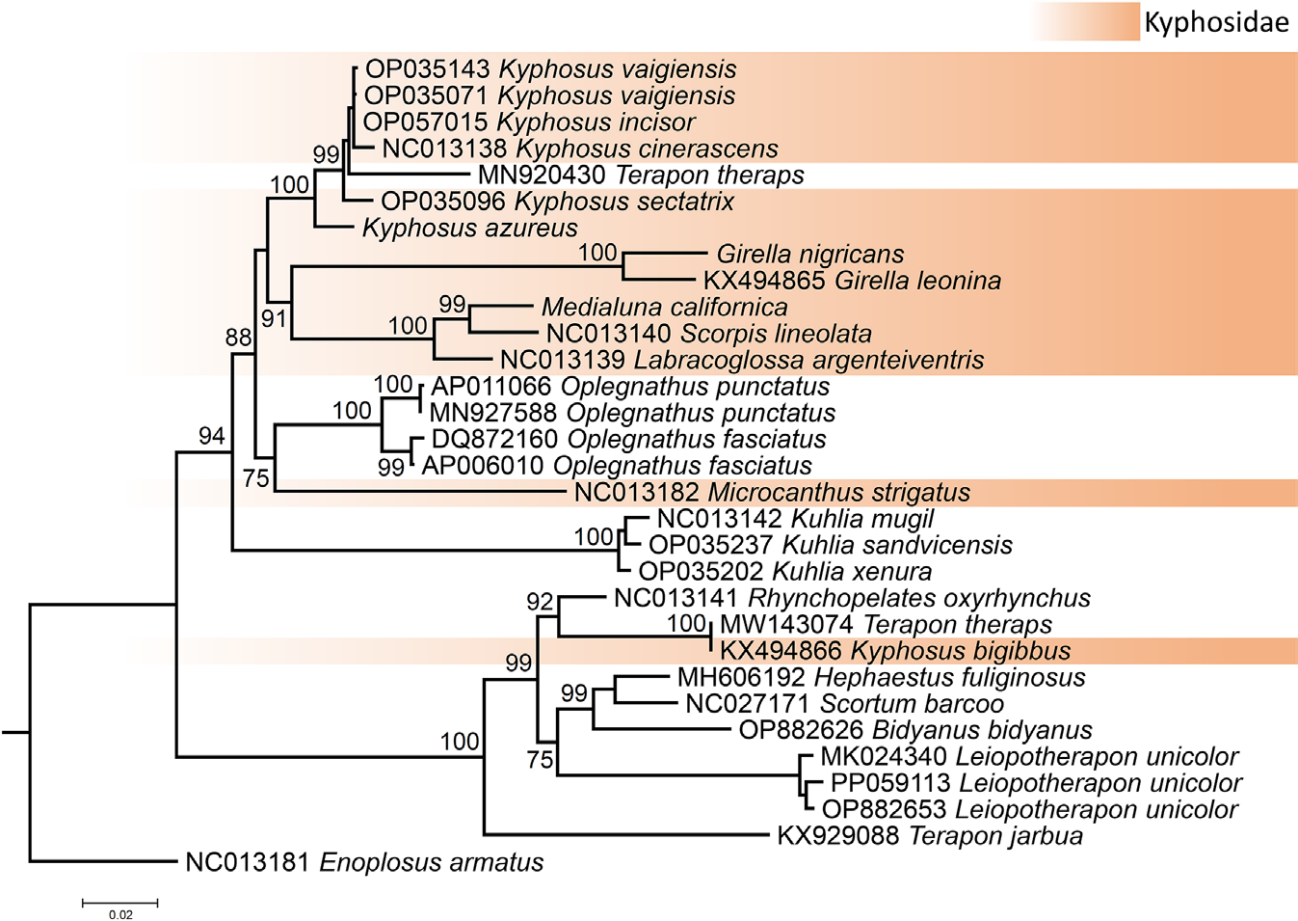
Mitophylogenomic analysis of the family Kyphosidae, including mitochondrial genomes of the species of sea chubs from the north eastern Pacific Ocean assembled with short reads. Values close to the nodes represent maximum likelihood bootstrap support values.

The ML phylogenetic analysis, based on 3,796 amino acid characters of which 829 were informative, did not support the monophyly of the family Kyphosidae taking into consideration that not all the specimens belonging to this family used in the analysis clustered together into a single supported clade (Figure 6). Specifically, a single specimen of *Kyphosus biggibus* sequenced by Hou et al. (GenBank accession number KX494866, unpublished data) clustered and formed a fully supported clade (bootstrap value [bv] = 100) together with nine specimens belonging to the family Terapontidae (Figure 5). Also, the Australian Stripey *Microcanthus strigatus* clustered together in a moderately supported clade with four specimens belonging to the family Oplegnathidae, the latter representing two species of *Oplegnathus*. Lastly, a specimen of *Terapon theraps* (MN920430) in the family Terapontidae clustered together into a fully supported clade with all other specimens of the genus *Kyphosus* used in the phylogenetic analysis except *K*. *biggibus*. A second mitochondrial genome of *T*. *theraps* sequenced by Zhou et al. (MW143074, unpublished data) clustered together (as expected) with other specimens belonging to the family Terapontidae + *K*. *biggibus*. Altogether, the aforementioned phylogenetic relationships suggest that the specimens sequenced by Hou et al. (KX494866) and Zhou et al. (MW143074) were misidentified; the specimen labelled as *K*. *biggibus* might be a representative of the family Terapontidae while the specimen labelled as *Terapon theraps* by Zhou et al. might be a representative of the genus *Kyphosus*.

Although our ML phylogenetic analysis did not support the monophyletic status of the family Kyphosidae, all specimens belonging to the genus *Kyphosus* (+ *T*. *theraps—*MN920430) clustered into a fully supported phylogenetic clade. In turn, *G*. *nigricans* clustered into a fully supported clade with *G*. *leonina*, the only other congeneric species used in the analysis, Lastly, *M*. *californiensis* formed a fully supported clade with *Labracoglossa argenteiventris* and *Scorpis lineolata* (Figure 6).

## 4 Discussion

In this study, full mitochondrial genomes were assembled using Illumina short reads in three species of sea chubs. These short‐read assemblies were regarded as ‘gold standards’ to benchmark the accuracy of de novo assembled mitochondrial genomes employing ONT long reads exclusively using very low (1×, genome skimming), low (3–5×) and high (> 25×) sequencing depth. Using the latest nanopore long‐read sequencing technology, alongside a specialised bioinformatics pipeline, we did assemble circular (complete) mitochondrial genomes in the three studied species when using high sequencing depth ONT data sets. Importantly, all of the long‐read assemblies using high sequencing depth (ONT) exhibited relatively high assembly coverages (39–94×) and two of the long‐read assemblies (belonging to *G*. *nigricans* and *K*. *azureus*) were ‘perfect’; they were identical to their short‐read assembly counterparts. Interestingly, the third ONT long‐read assembly belonging to *Medialuna californiensis* was 14 bp longer than the respective short‐read assembly. It could be argued that in *M*. *californiensis*, the disparity between the two assemblies is due to the failure of short reads to assemble short tandem repeats when present in mitochondrial genomes. Indeed, our observations are similar to those reported by a recent study that compared mitochondrial genomes assembled with PacBio HiFi long reads with short‐read mitochondrial genome assemblies for the same species. When PacBio assemblies are longer than their short‐read counterparts, repeats are the cause of the longer MitoHiFi assemblies (Uliano‐Silva et al. 2023). Importantly, we inspected a coverage‐to‐assembly‐length plot in the short‐read assembly of *M*. *californiensis* and we did not observe unusually high‐coverage peaks in the motif that was repeated twice in the ONT assembly. Coverage peaks are often indicative of collapsed tandem repeats (Douglass et al. 2019). Furthermore, none of the low sequencing depth (3× and 5×) ONT assemblies exhibited this replicated 14 bp motif, in line with the short‐read assembly. At present, we cannot argue that, in the case of *M*. *californiensis*, the long‐read, and not the short‐read assembly, is perfect, as claimed to be the case in PacBio assemblies when those are longer than its short‐read counterparts (Uliano‐Silva et al. 2023). Overall, our results suggest that high sequencing depth ONT long‐read mitochondrial genome assemblies are perfect most of the time but errors might be present in these assemblies.

Our subsampling scheme of ONT long reads demonstrated that at 3× and 5× subsampling, genomes were identical (perfect) or almost identical (quasiperfect) to their respective Illumina assemblies. Only a single (in *K*. *azureus* and *M*. *californiensis* with 5× and 3× sequencing depth, respectively) or a few errors (*n* = 11 on *G*. *nigricans* with 3× sequencing depth) were detected in the ONT low sequencing depth assembled mitochondrial genomes. However, read subsampling at a sequencing depth of 1× (considered genome skimming) resulted in the assembly of partial or complete mitochondrial genomes with numerous errors, including, among others, simple indels and indels at homopolymer regions. At very low sequencing depth (genome skimming), our results are somewhat similar to those reported by studies published a few years ago that assembled mitochondrial genomes with ONT long reads alone but employed older nanopore flowcells (R9) and chemistries. For instance, in the only two previous studies that have benchmarked the accuracy of ONT long‐read mitochondrial genomes with full reference genomes produced with short‐read Illumina sequencing (in the Caribbean spiny lobster *Panulirus argus* using ONT sequencing depth of 0.06×—Baeza 2020, in the silky shark *Carcharhinus falciformis* with ONT sequencing depth of 0.07×—Baeza and García‐De León 2022), numerous (*n* = 77–51 and 110–65 in *P*. *argus* and *C*. *falciformis*, respectively) insertions and/or deletions at the flanks of homopolymer regions, among other errors, were commonly reported both de novo‐ and reference‐based in the ONT assemblies after benchmarking them with short‐read assemblies. Insertions and/or deletions in homopolymer runs were also reported as the most frequent ‘errors’ in ONT assemblies by a few other studies that have analysed in detail error types in chloroplast (Scheunert et al. 2020) and bacterial genomes (Wick and Holt 2019) assembled with ONT long reads alone. In this study, the number of total errors was lower than in previous studies and simple insertions and deletions were more common errors than indels at the flanks of homopolymer regions as reported by previous studies. Our results suggest that the latest ONT platforms and chemistries exhibited decreased homopolymer errors and they can be used, exclusively, to de novo assembly complete and ‘perfect’ mitochondrial genomes when using high and low but not very low (=genome skimming) sequencing depth sequencing data sets.

We highlight the value of independent benchmarking of long‐read accuracy through the last years. The detailed report on error type by previous and this study has allowed us to record ONT long‐read improvements in a short time period. As the authors write this paper, ONT continues developing and testing new pores and chemistries that can produce raw reads with low initial error rates (see www.nanoporetech.com). As suggested by Baeza and García‐De León (2022), long‐read technology (at high sequencing depth) is currently able to assemble complete and totally accurate mitochondrial genomes. It remains to be addressed if longer genomes, including chloroplasts, plant mitochondrial genomes and bacterial chromosomes can be assembled reliably using ONT long reads exclusively (Wick and Holt 2019). Overall, this study demonstrates that perfect (complete and fully accurate) or quasiperfect (complete but with a single or a very few errors) mitochondrial genomes can be assembled using high (> 5×)‐ and low‐(3–5×)‐coverage ONT long read exclusively, but not at very low‐coverage sequencing depths (genome skimming data sets at 1×) sequenced using the latest platforms and chemistries. We note that we used blood for gDNA extraction in the studied species. The use of mitochondrial‐rich tissues (i.e., cardiac muscle, extraocular muscles and or liver, compared to blood, see Liao et al. 2020 and references therein) is likely to result in increased mitochondrial genome assembly coverage using the same strategy used in this study, ultimately permitting further decreases in the cost of mitochondrial genome sequencing.

### 4.1 Mitochondrial Genome Organisation in Sea Chubs

We have characterised in detail the mitochondrial genomes belonging to three studied sea chubs. Gene content and organisation in the studied mitochondrial genomes were identical to those reported for cofamilial species (Yagishita et al. 2009; Kim et al. 2020) and other fishes in the order Perciformes (Molina‐Quirós, Hernández‐Muñoz, and Baeza 2022). Also, the total length of the newly assembled mitochondrial genomes is very similar to those of congeneric and cofamilial species. In the family Kyphosidae, mitochondrial genomes vary in total length between 16,492 bp in *Labracoglossa argentiventris* (AP011062—Yagishita et al. 2009) and 16,542 bp in *K*. *vaigiensis* (OP035071) and *K*. *incisor* (OP057015—no companion paper).

All three newly assembled mitochondrial genomes were A + T rich, in line to that observed in other cofamilial species in which estimated A + T content varies between 53.09% in *Scorpis lineolata* (NC013140—Yagishita et al. 2009) and 56.26% in *Girella leonina* (KX494865). The A + T rich nature of mitochondrial genomes, including those studied herein, remains poorly understood, but it might be explained, among others, by differences in the efficiency of selection for different nucleotides, codon usage bias (with a preference for codons containing A, T, or both), mutational bias, or a combination of the above (Long et al. 2018).

tRNA genes in the mitochondrial genomes of *G*. *nigricans*, *K*. *azureus* and *M*. *californiensis* were similar in length to those of other teleost fishes (Satoh et al. 2016). All tRNA genes exhibited the standard ‘cloverleaf’ secondary structure as predicted by MitFI except tRNA‐Ser1 that lacked the D‐arm loop. A truncated tRNA‐Ser1 gene has also been reported for 250 other teleost fishes in which the secondary structure of tRNA genes has been studied (Satoh et al. 2016). Indeed, the absence of the D‐arm loop, stem or entire D‐arm in the mitochondrial tRNA‐Ser1 gene seems to be conserved across eumetazoans and though it is truncated, this gene has been proposed to be functional (Watanabe, Suematsu, and Ohtsuki 2014).

The few previous studies characterising the mitochondrial genomes in representatives of the family Kyphosidae have not examined the organisation of the CR. Nonetheless, microsatellites, tandem repeats and ‘hairpins’, as observed in the CR of the studied species, are common in the CR of most fish (Molina‐Quirós, Hernández‐Muñoz, and Baeza 2022). We argue in favour of additional studies comparing the organisation of this region in this and other fish clades to improve the understanding of the function of the CR during mitochondrial transcription and replication (Bernt, Bleidorn, et al. 2013).

Overall, the similarities observed among the studied mitochondrial genomes, all belonging to species in the family Kyphosidae, and those of closely related species highlights the highly conserved function of mitochondrial genomes despite remarkable ecological and physiological disparity in fish (Bernt, Bleidorn, et al. 2013). The observed organisation of the newly assembled mitochondrial genomes using ONT long reads (at high sequencing depth) represents another line of evidence supporting the notion that the assembled ONT mitochondrial genomes are fully accurate.

This study assembled for the first time mitochondrial genomes belonging to three common sea chubs in the coast of the northeastern Pacific Ocean that is currently experiencing frequent heat wave events in addition to more localised environmental challenges, such as habitat alteration and pollution (Shanks et al. 2020, and references therein). The assembled and annotated (in detail) mitochondrial genomes, together with others already available in NCBI's GenBank, can provide a baseline for future studies focusing on the conservation and fisheries management of these species. For instance, the newly assembled mitochondrial genomes can be used as a reference in environmental DNA studies focusing on bioprospecting and biomonitoring of these and other coastal species before and after heat waves or experiencing environmental challenges.

### 4.2 Mitophylogenomics of Sea Chubs

In general, the recovered phylogenetic relationships are in line with previous studies that have not provided support for the monophyletic status of the family Kyphosidae but which have used a smaller set of mitochondrial genomes (e.g., Kim et al. 2020) or a larger number of species but with a smaller set of molecular markers (e.g., Knudsen and Clements 2016) for phylogenetic reconstruction compared to this study. Our analysis also suggests misidentification problems in specimens used for the assembly of mitochondrial genomes. We argue in favour of additional studies focusing on the sequencing, assembling and detailed characterisation of mitochondrial genomes to continue improving the understanding of the evolutionary history of this remarkable clade of mostly herbivorous fishes. Expanding the mitochondrial genome reference database for teleost fishes will improve our ability to tackle conservation and management problems affecting them, including surveillance and detection of mislabelling and/or misidentification of these commodities in the supply chain and market place (e.g., Baeza and Behringer 2017).

### 4.3 Conclusion

This study assembled full and completely accurate mitochondrial genomes in three species of sea chubs using the latest ONT long‐read sequencing technology at high sequencing depth. Other than demonstrating that full and completely accurate mitochondrial genomes can be assembled using the latest ONT long‐read sequencing technology, we argue that ONT sequencing technology, given the size of the sequencing device (pocket‐sized) and cost of consumables (cheap [cost per Gb < USD 50] compared with other platforms [> USD 600 per Gb], see Table S4 for a cost comparison among short‐read and long‐read platforms), can be potentially used to improve access to high‐throughput sequencing technologies in low‐ and moderate‐income countries (and in the ‘Global South’). Based on the assembly result we show here (5× nuclear genome coverage for zero mitochondrial assembly errors), and the average throughput (in our hands) of the handheld ONT MinION (~20 Gb), up to four samples could be barcoded to achieve perfect mitochondrial sequencing. In line with the idea of democratising sequencing and bioinformatic analyses, we have run our pipeline for the assembly of mitochondrial genomes with ONT long reads exclusively in the Galaxy platform (The Galaxy Community 2022), and thus, researchers in low‐ and moderate‐income countries can replicate the analyses at minimal cost.

## Author Contributions

J.A.B. conceived and designed the experiments. J.J.M. carried out the collections, molecular biology, sequencing and analysis of the fish samples. J.A.B. carried out the bioinformatic analysis and wrote the manuscript. J.A.B., J.J.M. and T.P.M. supervised the project.

## Ethics Statement

No human data were used in this study. Animal samples were donated by local fishers and sample processing under IACUC Animal Use Protocol S12219. No approval by an ethical committee was required to accomplish the goals of the present study because experimental work was conducted with specimens retrieved from local fishermen.

## Consent

The authors have nothing to report.

## Conflicts of Interest

The authors declare no conflicts of interest.

## Benefit‐Sharing Statement

A research collaboration was developed between scientists in California and South Carolina. Scientists in California providing genetic samples, all collaborators are included as coauthors, and the research addresses a priority concern, in this case the conservation of organisms being studied. More broadly, our group is committed to national and international scientific partnerships, as well as institutional capacity building. Benefits Generated: Benefits from this research accrue from the sharing of our data and results on public databases as described above.

## Supporting information


Data S1.

## Data Availability

Raw sequence reads are deposited in the SRA (Accession numbers: SRR28380136 [*G*. *nigricans*], SRR28380135 [*K*. *azureus*] and SRR28380134 [*M*. *californiensis*]). All data sets on which the conclusions of the manuscript rely are available at the SRA database of NCBI with Bioproject number PRJNA971989.
