# Supplementary material for: Assembly of Mitochondrial Genomes Using Nanopore Long‐Read Technology in Three Sea Chubs (Teleostei: Kyphosidae)
Source: Mol Ecol Resour. 2024 Oct 15;25(1):e14034. doi: 10.1111/1755-0998.14034 (PMC11646298; doi:10.1111/1755-0998.14034)
Supplement: Supplementary file 1 — Data S1. [file MEN-25-e14034-s001.docx]

**Perfect and quasi-perfect assembly of mitochondrial genomes using the latest nanopore long-read technology at high- and low- but not at genome skimming (1x) sequencing depth in three sea chubs (Teleostei: Kyphosidae) from California, USA**

J. Antonio Baeza^1, 2, 3,*^, Jeremiah J. Minish^4^, Todd P. Michael^4^

*^1^ Department of Biological Sciences, 132 Long Hall, Clemson University, Clemson, SC 29634, USA.*

*^2^ Smithsonian Marine Station at Fort Pierce , 701 Seaway Drive, Fort Pierce, Florida 34949, USA.*

*^3^ Departamento de Biología Marina, Facultad de Ciencias del Mar, Universidad Católica del Norte, Larrondo 1281, Coquimbo, Chile.*

*^4^ The Plant Molecular and Cellular Biology Laboratory, Salk Institute for Biological Studies, 10010 N. Torrey Pines Rd., La Jolla, CA, 92037, USA*

^*^correspondence: JA Baeza, jbaezam@clemson.edu, phone:1 864 – 973 - 9157

**Supplementary Materials**

**Supplementary Table S1.** Microsatellites present in the mitochondrial control region of *Girella nigricans*.


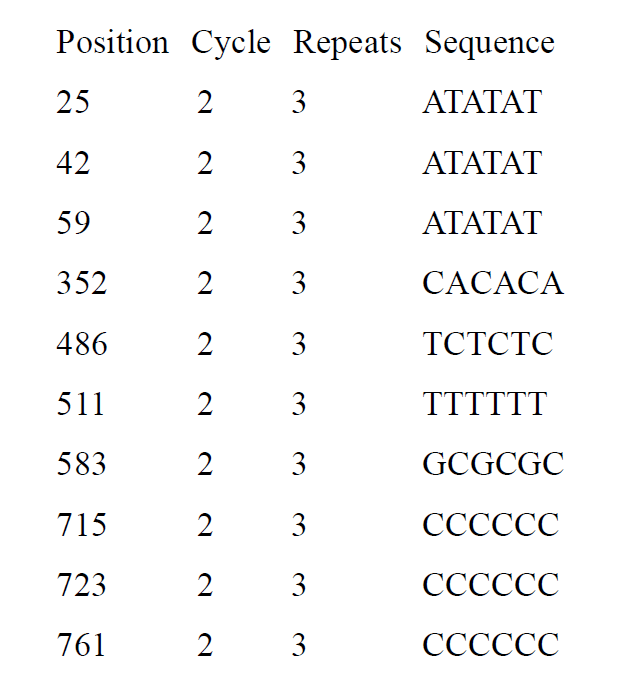


**Supplementary Table S2.** Microsatellites present in the mitochondrial control region of *Kyphosus azureus*.


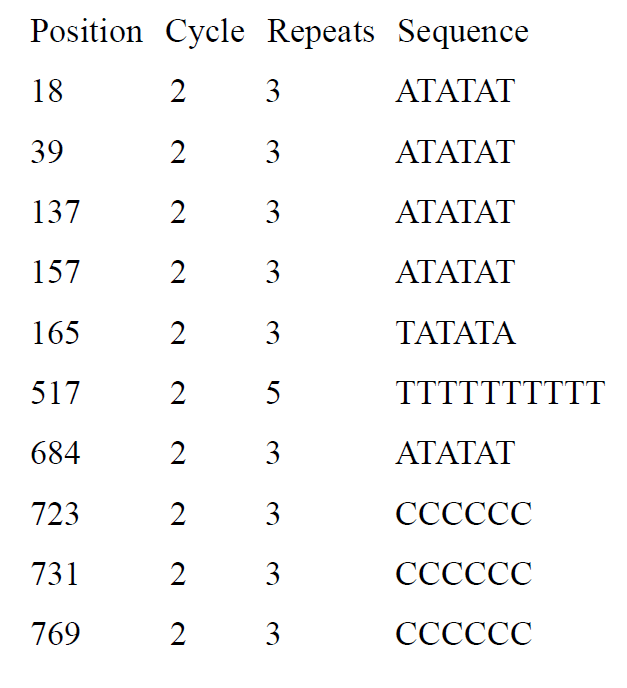


**Supplementary Table S3.** Microsatellites present in the mitochondrial control region of *Medialuna californiensis*.


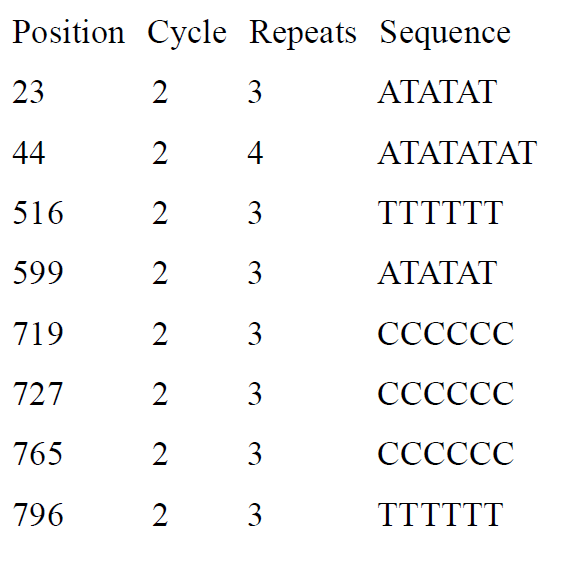


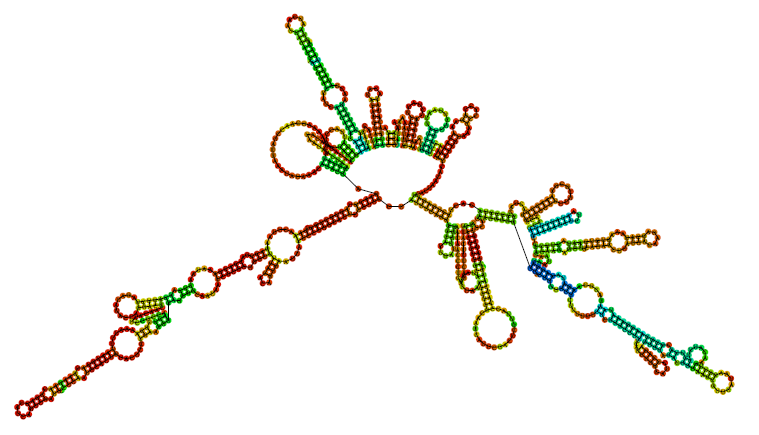


**Supplementary Figure S1.** Secondary structure of the mitochondrial control region of of *Girella nigricans*.


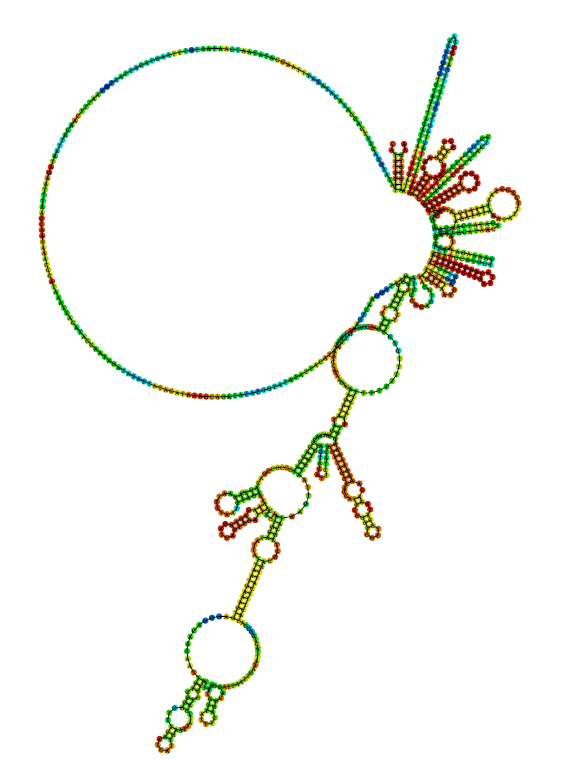


**Supplementary Figure S2.** Secondary structure of the mitochondrial control region of *Kyphosus azureus*.


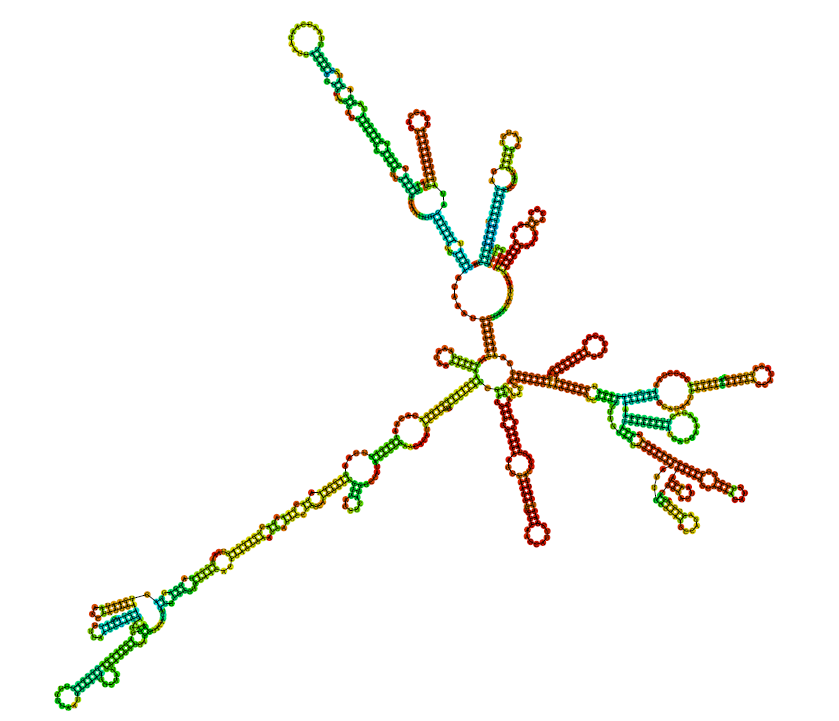


**Supplementary Figure S3.** Secondary structure of the mitochondrial control region of *Medialuna californiensis*.

**Supplementary Table S4.** Cost of the different platforms used for NGS currently (long and short reads). Only the cheapest devices from Illumina and ONT are compared.

|  | Illumina NextSeq 2000 | PacBio  Revio | ONT  Minion |  |
| --- | --- | --- | --- | --- |
|  |  |  |  |  |
| Platform Cost | USD 335,000 | USD 779,000 | USD 2,000 |  |
| Flow Cell Cost | USD 2,890  (200 cycles, P4 flow cell) | USD 995  (SMRT Cell) | Included with ONT minion start package (USD 450) |  |
| Output (Yield) | 540 Gb | 90 Gb | 48 Gb |  |
| Cost per 1 Gb | USD 625.72 | USD 8,666.61 | USD 41.67 |  |
|  |  |  |  |  |
|  |  |  |  |  |

Costs of small equipment is not included as it is expected to be available in genetics / genomics laboratories.
